# Do We Stand Together? The Role of Perceived Personal and Group Threats in Predicting the Majority’s (Un)willingness to Confront Injustice on Behalf of a Minority

**DOI:** 10.3389/fpsyg.2021.694044

**Published:** 2021-07-19

**Authors:** Göksu Celikkol, Inga Jasinskaja-Lahti, Tuuli Anna Renvik, Raivo Vetik, David Lackland Sam

**Affiliations:** ^1^Department of Social Research, University of Helsinki, Helsinki, Finland; ^2^Open University, University of Helsinki, Helsinki, Finland; ^3^School of Governance, Law, and Society, Tallinn University, Tallinn, Estonia; ^4^Department of Psychosocial Science, University of Bergen, Bergen, Norway

**Keywords:** realistic threats, group threats, perceived discrimination, economic insecurity, collective action, minority rights

## Abstract

**Purpose:** By utilizing data from Estonia, Finland, and Norway, this study explores how the perceptions of personal and group realistic threats, namely perceived ethnic discrimination and economic insecurity among national majorities, predict their unwillingness to confront injustice on behalf of Russian-speaking minority groups.

**Background:** Previous research on collective action to promote minorities’ rights and social standing has focused either on minorities’ own actions or factors promoting the willingness of majority group members to engage in collective action on behalf of minorities. In contrast, factors explaining the reluctance of majority group members to engage in collective action on behalf of minority groups have remained less explored. For example, studies have then ignored that the majority members may also feel threatened and may be economically insecure. Furthermore, the possible discrepancy between perceived personal vs. in-group’s situation may influence majority group members’ (un)willingness to confront injustice on behalf of a minority group.

**Method:** We employed polynomial regression with response surface analysis to analyze data gathered among national majority members in three countries (*N* = 1,341).

**Results:** Perceived personal and group realistic threats were associated with heightened unwillingness to confront injustice on behalf of the Russian-speaking minority. Furthermore, participants were more unwilling to confront injustice when they perceived more group than personal threat.

**Conclusion:** We found that majority group members’ (un)willingness to confront injustice on behalf of the minority is related to how secure they perceive their own and their group status. Our results contribute to previous research by pointing out the important drawbacks of majorities’ support for minorities’ wish for social change.

## Introduction

Previous research on support for collective action, i.e., actions and responses that aim to improve the position and status of an in-group, has often focused on the attitudes, intentions, and behaviors of disadvantaged and low-status groups, which usually also represent numerical minorities (see, e.g., Wright et al., 1990; van Zomeren et al., 2008). Such efforts are crucial to make the voices of minorities heard and to combat social inequalities. Inequality cannot, however, be seen as a matter of concern for only those who are most exposed to its negative consequences. For social change to occur, the efforts of majority groups are decisive. Not only do majority group members have more power (Sidanius and Pratto, 1999; Mallett et al., 2008), their actions taken on behalf of other groups in the society can be perceived as more persuasive than collective action by minority group members themselves (e.g., Mallett et al., 2008). These notions speak for the need for minority groups to have the support of the majority to better succeed in collective actions and, consequently, for social change to happen.

Indeed, there is evidence of social movements and protests that gained support from not only the disadvantaged groups but also from the advantaged majority group members, even though the advantaged group members themselves did not directly benefit from it (see, e.g., Subašić et al., 2008). Jetten (2019) recently pointed out that advantaged group members are more likely to support disadvantaged groups when group boundaries are seen as permeable, intergroup relations are perceived as secure, and the in-group’s position is regarded as legitimate and stable (i.e., “noblesse oblige,” see also Mols and Jetten, 2017). However, if an advantaged group assumes its superior position as legitimate but insecure, it may be highly discriminatory to secure its interests (Turner and Reynolds, 2001; see also Jetten, 2019).

In the context of immigration, there is increasing evidence that national majority groups, including their most wealthy members, often perceive immigration to pose a threat to their identity, security, and material wellbeing, and this may further heighten anti-immigrant sentiments (Schmuck and Matthes, 2014; Jetten et al., 2015; Shepherd et al., 2018; Hasbún López et al., 2019). Thus, majority group members may perceive minority groups’ social mobility as challenging the status quo in which the majority group has a higher status, more resources, and privileges (Jetten, 2019). Hence, withdraw their support from actions promoting minority’s position within the society or even engage in collective action against minorities (Hasbún López et al., 2019).

Research suggests that advantaged group members’ support for system-challenging collective action is particularly not likely to occur in the presence of a perceived threat to their privileged position within the society (e.g., Stefaniak et al., 2020). Cottrell and Neuberg (2005) argued that such threat perceptions include threats to physical safety, group possessions and resources, and personal freedom. Integrated threat theory (Stephan and Stephan, 2000) defines perceived threats to in-group’s economic situation and power, existing resources, and their security and existence in general as perceived realistic threats. Accordingly, previous research shows that national majority group members often perceive not only economic insecurity (see Jetten et al., 2015) but also social insecurity (i.e., feelings of insecurity in social situations) as a result of the prejudice and discrimination that they perceive from the ethnic minorities and immigrants (Kluegel and Bobo, 2001; Norton and Sommers, 2011). Both threats explain their opposition to immigration (e.g., Ferwerda et al., 2017; Jetten, 2019; Hasbún López et al., 2019; Wright and Esses, 2019). In the present study, we focus on two types of perceived realistic threats from the viewpoint of national majority group members: perceived ethnic discrimination and economic insecurity.

It is important to note that while research has examined various types of threats as experienced by majority group members in relation to immigrants, experiences of ethnic discrimination as a form of perceived threat among the majority members have received little research attention. There are, however, notions of the so-called “reverse discrimination” where majority members experience affirmative action policies (Bergmann, 1999) or diversity policy messages in organizations (Dover et al., 2016) as favoring members of disadvantaged groups (Norton and Sommers, 2011). Previous research found that such perceptions are linked with threats related to perceived changes in the racial status quo and increased outgroup bias (Wilkins et al., 2017). Also, more directly measured experiences of being discriminated against have been shown to go hand-in-hand with majority group members’ feelings of insecurity, vulnerability, and deprivation, leading to increased anti-immigration rhetoric (see, e.g., Inglehart and Norris, 2016; Spruyt et al., 2016) and heightened perceptions of competition over scarce resources (e.g., Esses et al., 1998). Thus, we argue that perceptions of both realistic threats economic insecurity and discrimination may make advantaged majority group members withdraw from actions that benefit minorities.

Moreover, it is crucial to recognize that intergroup attitudes and behaviors are a reaction to a situation that is perceived as unfair (Runciman, 1966; Jasinskaja-Lahti et al., 2009) and/or threatening (Tajfel and Turner, 1979; Cottrell and Neuberg, 2005; Verkuyten, 2009) not only personally but also, or even more so, collectively. While the revised threat theory (Stephan and Renfro, 2002) and previous research have differentiated between perceived personal and group threats and disadvantage (Taylor et al., 1990; Postmes et al., 1999; Stephan and Renfro, 2002), to our knowledge, there are no previous studies on the simultaneous effects of perceived threats to personal and in-group’s security or economic status, when examining advantaged group members’ unwillingness to confront injustice on behalf of the minority.

Finally, previous research often links perceptions of disadvantage to outgroup negativity (Runciman, 1966; for a meta-analysis, see Smith et al., 2012). Yet, recently emerging research on relative gratification suggests that the relationship between perceptions of wealth and prejudice can be non-linear (i.e., those who are economically secured may show high levels of prejudice; Dambrun et al., 2006). In this study, we account for the complexity and the possible non-linearity of the relationship between perceived realistic personal and group threats and unwillingness to confront injustice on behalf of the minority (Shanock et al., 2010).

### Majority’s (Un)willingness to Support Disadvantaged Minorities

It is not uncommon that collective action which aims to improve the position of minority groups gets considerable support not only from members of other underprivileged (e.g., Brysk and Wehrenfennig, 2010) but also advantaged groups (e.g., Subašić et al., 2008; van Zomeren et al., 2011). The question of what motivates advantaged group members to engage in actions promoting the position of the disadvantaged has therefore received increasing interest in recent studies. For example, outgroup perspective-taking and group-based guilt (Mallett et al., 2008), perceived violations of moral convictions (van Zomeren et al., 2011), and common superordinate group identification (Vezzali et al., 2015) have been identified as possible mechanisms that facilitate majority group members’ collective action in support of disadvantaged minority groups. In addition, the positive intergroup contact has the potential to increase the willingness of majority group members to stand up for minority rights (Bagci and Çelebi, 2017; Reimer et al., 2017), in the form of ss stereotypical and negative evaluations of the outgroup (Kotzur et al., 2019).

Another line of research has focused on what makes advantaged group members withdraw from collective action on behalf of the disadvantaged. From a social identity theoretical point of view, advantaged group members are likely to take a harsh or even an oppressing stance toward the disadvantaged, if they see group boundaries as permeable, as it is possible to move between groups, and/or the in-group’s position as insecure (Jetten, 2019). Indeed, Jackson and Esses (2000) argued that majority group members’ wish to maintain their high status and power in society might undermine their willingness to support the empowering of the immigrants. They also documented that perceived economic competition and power struggle predict reluctance to support immigrants’ empowerment. Other research supports the critical role of perceived intergroup threats in collective action mobilization. For example, whereas perceptions of low levels of economic and cultural threats predicted more willingness to help immigrants in Burhan and van Leeuwen’s (2016) study, perceived intergroup threats predicted less willingness among majority group members to engage in collective action in support of the disadvantaged ethnic minority and immigrant groups in Shepherd et al.’s (2018) study. A recent 11-country European study by Hasbún López et al. (2019) also found more willingness to engage in collective action against refugees. Similarly, Stefaniak et al. (2020) have found that the increased attribution of the gains of the outgroup to the losses of the in-group (zero-sum game, Esses et al., 1998; Norton and Sommers, 2011) might not only prevent the advantaged group members from becoming allies in system-challenging collective action but increase their support for system-supporting collective action.

Typically, groups with a higher status within the society tend to have easier access to the resources than disadvantaged minorities. Thus, perceiving the advantaged in-group’s position as insecure might come with economic concerns. According to realistic conflict theory (Sherif, 1966; LeVine and Campbell, 1972), perceived competition over scarce resources can lead to conflict between two groups. Feelings of economic stagnation (Jetten, 2019) or relative deprivation (Runciman, 1966) may arise when a person feels that (s)he or her/his in-group should be entitled to something better or something that another person (or an outgroup) has. This may result in intergroup negativity. For example, the migration of both low- and high-skilled immigrants has been shown to evoke threat perceptions among national majorities. Immigrants that are of similar skill levels raise concerns among national majority members due to their ability to compete in the labor market (see Mayda, 2006). In contrast, immigrants of lower socioeconomic status may be considered economic burdens by national majority members due to their use of benefits, such as free healthcare and unemployment compensation (Boeri et al., 2002; Mayda, 2006). Therefore, as Walsh and Tartakovsky (2021) argued, majority groups’ perceptions of intergroup threats depend on the social structural characteristics and stereotypes of the groups in society. Importantly, not only the economically disadvantaged but also relatively well-off people can take a negative stance toward the disadvantaged, as they have more to lose, and as they might feel entitled to their advantaged position (Grofman and Muller, 1973; Guimond and Dambrun, 2002; Dambrun et al., 2006; Jetten et al., 2015).

Another concern among national majorities related to immigration is increased feelings of being a target of the minority’s prejudice and subsequent insecurity (Kluegel and Bobo, 2001; Norton and Sommers, 2011). There is research showing that also advantaged national majority groups report ethnic discrimination and that it is associated with their negative attitudes toward immigrants and immigration (e.g., Celikkol et al., 2017). According to Grigoryev et al. (2020), particularly those majority group members who have a negative outlook on the social world by perceiving it as dangerous and competitive (cf. Dual Process Model; Duckitt, 2001), and thus, score high in right-wing authoritarianism and social dominance orientation, respectively, tend to regard immigration, multicultural ideology, and intergroup contact as threatening. However, the relationship between perceived prejudice and discrimination stemming from the minority group and the majority group’s (un)willingness to support minorities may also be the opposite way. For example, group empathy theory posits that intergroup empathy (i.e., empathy felt by members of one group toward another group) can boost support on behalf of the other group even when the groups are in direct competition for rights, security, and resources (Sirin et al., 2016). Importantly, not only do different minority groups feel empathy toward each other, it is empathy that fuels their solidarity and mobilizes them to collective action. Additionally, majority group members may empathize with minority groups when they co-experience the unfair treatment experienced by minorities. Therefore, in this study, we argue that it is fruitful to study experiences of discrimination from the viewpoint of the advantaged, as this may account for advantaged group members’ (un)willingness to confront injustice on behalf of minorities.

Altogether, previous research indicates that national majority group members’ perceptions of realistic threats may evoke a need to maintain the current social order (e.g., Li, 2019), leading them to withdraw support from minority movements that aim to change the status quo within the society. In this study, we investigate how realistic threat perceptions in the form of ethnic discrimination and economic insecurity are associated with advantaged groups’ (un)willingness to confront injustice for the disadvantaged.

### Personal and Group Evaluations of Perceived Discrimination and Economic Insecurity

It is essential to acknowledge the possible discrepancies between personal and in-group’s experiences of disadvantage, as pointed out by Taylor et al. (1990) in their theorization of personal/group discrimination discrepancy (PGDD). They suggested that people tend to perceive their in-group to be disadvantaged or discriminated more than themselves as individuals (PGDD; Taylor et al., 1990). According to Crosby (1984), such discrepancy may result from denial or minimization of personal disadvantage. Later on, Taylor et al. (1990) argued that it might be the other way around, i.e., the minority group members may exaggerate the level of discrimination that is targeted to their group in order to increase the probability of collective actions that may improve or maintain the in-group’s status (see also Taylor et al., 1994). Finally, Quinn et al. (1999) proposed that such discrepancy is a result of the relative informational complexity involved in the comparison targets, with personal ratings being influenced by personal judgments and group ratings by social motives (Taylor et al., 1994; Postmes et al., 1999). In fact, it has been argued that in line with the social identity theory (SIT; Tajfel and Turner, 1979), it is especially beneficial for people to see themselves as more privileged and better-off than the other group members. This is a form of coping mechanism or a personal protection strategy (Smith and Spears, 1996; Postmes et al., 1999). In their study, Postmes et al. (1999) used a general disadvantage rating instead of discrimination and obtained evidence that such discrepancy not only occurs for discrimination – participants reported lower personal disadvantage compared to their group.

It should be noted here that both the cognitive and SIT and research on personal and group discrimination discrepancy often focus on the disadvantaged minorities. Yet, such discrepancy is visible regardless of the status of the group within the society, and there is a small but growing body of research showing that personal-group discrepancy in discrimination applies to both underprivileged and privileged groups (Postmes et al., 1999). Similarly, Celikkol et al. (2017) found that Finnish majority group members in Finland perceived higher levels of group discrimination than personal discrimination.

The distinction between personal and group perspectives has also been made within relative deprivation theory (Runciman, 1966) that distinguishes fraternalistic (group) deprivation from egoistic (personal) deprivation and suggests that these two types of deprivation may lead to distinct consequences (see Guimond and Dubé-Simard, 1983; Dube and Guimond, 1986). It has been argued that group-level (but not personal-level) comparisons are a predictor of social behavior (see Walker and Pettigrew, 1984) and prejudice (see Pettigrew et al., 2008; Celikkol et al., 2017). Furthermore, previous research suggests that an individual may have a positive view of his/her own living conditions, wellbeing, and lifestyle while having a negative view of the situation in the country in general (Whitman, 1998). While the present research does not focus on relative deprivation, it acknowledges that people may have concerns about their personal economic situation as well as their in-group’s economic standing. Moreover, such concerns are not specific to the economically disadvantaged groups in society only. For example, Vanneman and Pettigrew (1972) found that Whites who felt fraternally deprived had the highest reluctance to support Black political candidates.

To sum up, previous research highlights the critical role of the perceptions of personal and group realistic threats (in terms of economic insecurity and ethnic discrimination) in explaining the majority’s unwillingness to confront injustice on behalf of minorities. However, it fails to show how personal and group threats perceptions are jointly and non-linearly linked to intergroup outcomes. Therefore, in this study, we examine whether and how not only the joint (or additive) effect of these two types of threats but also the possible discrepancies (including non-linear associations) between them account for majority group members’ (un)willingness to confront injustice.

### Aims and Hypotheses

In this three-country study conducted in Estonia, Finland, and Norway, the main aim was to complement previous research by showing how national majority group members’ perceptions of personal and in-group’s realistic threats (economic insecurity and ethnic discrimination) may explain unwillingness on their part to confront injustice on behalf of the Russian-speaking minorities. All three countries share a border with Russia and have a sizeable Russian-speaking immigrant population. Additionally, even though in all these three countries, Russians are known to face high levels of prejudice (e.g., Jaakkola, 2000; Aure, 2011; Varjonen et al., 2017; Vetik, 2019), their integration prospects are relatively good compared to other immigrant and refugee groups due to the Russians high level of education, cultural closeness, transnational networks, and economy (e.g., Varjonen et al., 2017). We argue that in the context of increasing immigration, majority group members may feel threatened by voluntary immigrants and the diasporic immigrant communities in their countries. They may have feelings of not being “better-off” enough or being targets of unfair integration policies that favor minority group members. They may also perceive ethnic prejudice (see Turner and Reynolds, 2001; Jetten, 2019), and these perceptions may result in their reluctance to confront injustice on behalf of the disadvantaged.

Methodologically, we use a novel statistical approach that is only emerging within the social sciences field, namely polynomial regression with surface response analysis (Shanock et al., 2010). This approach allows us to see the predictive power of the different combinations of personal and group disadvantages. Following the argument by Denissen et al. (2018), we see many advantages in preferring response surface analysis over traditional analytical approaches in our study. Such analysis helps us see how congruence (or agreement) between two predictors is related to the outcome and demonstrates how the discrepancy (or disagreement) between the predictors can be related to the outcome. With polynomial regression analysis, it is also possible to see whether it is agreement or disagreement between variables that better predicts the outcome and whether the relationship is linear or non-linear.

Intergroup threat theory (Stephan and Stephan, 2000; Stephan and Renfro, 2002) distinguishes between personal and group threats, personal-group discrepancy research (e.g., Taylor et al., 1990) posits the gap between personal and group evaluations of injustice, and SIT-based research on collective action (e.g., van Zomeren et al., 2008) recognizes the important role of perceived injustice and threat on collective action intentions. Based on these research lines, two different predictions for the association between majority group members’ perceived disadvantage and their (un)willingness to confront injustice on behalf of a minority can be made. On the one hand, we could expect that the higher perceptions of both personal and group threats will predict majority members’ unwillingness to confront injustice. This prediction is based on the notion that when one perceives threats on several dimensions, i.e., both personally and for fellow in-group members, it is more likely that this multifaceted threat perception evokes defensive intergroup reactions. Previously, it was argued that experiencing disadvantage in more than one dimension in life may exacerbate the negative effects of disadvantage (DiPrete and Eirich, 2006; Renvik et al., 2018), a phenomenon referred to as having a cumulative disadvantage. Experiencing multifaceted, cumulative disadvantages in the form of personal- and group-level realistic threats might make people more reluctant to help the outgroup perceived as the source of these threats. Consequently, our first hypothesis is follows:

*H1*: National majority group members, who perceive higher levels of personal and group threats (in terms of ethnic discrimination, H1a, and economic insecurity, H1b), will be more unwilling to confront injustice for Russian-speaking minority group members.

Based on perceived PGDD research (Taylor et al., 1990), people tend to perceive more discrimination directed to their in-group than themselves. Importantly, as Tajfel and Turner (1979, p. 34) argued, *intergroup* reactions result from perceived conflict between *groups*. Moreover, as Sassenberg and Woltin (2009) discussed, group members self-regulate based on their social identity, which means that the outcomes of one’s behavior are evaluated based on their effects on the in-group as a whole rather than for the individual. Therefore, heightened levels of outgroup negativity may arise even as a reaction to perceived personal advantage in the context of group disadvantage. In this study, we explore whether it is the perceptions of doing personally, economically better while perceiving the in-group to be doing worse, as well as not being personally discriminated but perceiving the in-group to be discriminated may also cause (i.e., the discrepancy between the low- or under-estimated perceived personal and high- or overestimated group threats) that predicts the majority members’ higher willingness to protect their group interests by showing less support for minorities. Consequently, our second hypothesis is follows:

*H2*: Those who perceive higher levels of threat to their in-group as a whole and lower levels of threat to themselves (H2a for ethnic discrimination and H2b for economic insecurity) will be more unwilling to confront injustice on behalf of the minority group.

While both hypotheses H1 and H2 predict a linear association between the personal/group threat perceptions and (un)willingness to confront injustice on behalf of the minority, based on the research and growing evidence on relative gratification and V-curve hypothesis (Dambrun et al., 2006; Jetten et al., 2015), we should also account for possible non-linear relations between our predictor variables and the outcome. Therefore, given the advantage of using polynomial regression analysis, we explore possible non-linear relations between the perceived personal/group realistic threats and willingness to confront injustice in this study.

Although reactions to the same minority group are examined in three neighboring countries with many similarities that we discuss next, we also account for the contextual differences, for example, in the size and societal position of Russian-speaking minorities. Thus, while our primary focus is on the general social-psychological mechanism related to perceived personal and group threats as predictors of unwillingness to support minority outgroups, we also explore possible between-country differences (RQ3).

### Contexts of the Study

Estonia regained its independence after the collapse of the Soviet Union in 1991. Although Russians have been living in Estonia for centuries, it was only at the start of the Soviet period, the Russian-speaking minority was known to make up the largest minority group in Estonia, constituting around 25 percent of the whole population. After gaining its independence, Estonia adopted the Citizenship Act in 1993 as a part of the new Estonian citizenship law, which required Russians who have arrived in Estonia during the Soviet era to go through a naturalization process that required competence in the Estonian language. As a result, a large group of Russians has become stateless (Barrington, 1995). Even today, around 80.000 stateless people live in Estonia, and approximately half of the Russians hold Estonian citizenship (Vetik, 2019). Furthermore, during the Soviet era, several political institutions in Estonia operated in Russian, and a separate Russian language education system was established. Russians who arrived in the country did not see the need to learn Estonian until the new citizenship law (see Kruusvall et al., 2009). Therefore, Estonia’s language reform harmed Russians’ status in the labor market and their education. This was perceived as “a form of forced acculturation or a vehicle for exclusion, aiming to create a unitary nation-state in Estonia” (Kruusvall et al., 2009) by the Russian minority. On the other hand, Russia is often perceived as a nearby threat in Estonia. Any protests against the new citizenship policy were perceived as directly against Estonia and as a representation of the imperialist desires of Russia (Vetik, 2019).

In Finland, Russian-speaking immigrants are one of the largest immigrant groups (Statistics Finland, 2019) and one of the oldest ethnic minorities dating back to times when Finland was under the rule of the Russian Empire. Finland gained independence in 1917, but with the start of the Winter War in 1939–40 between Finland and the USSR, it lost some of its territories. Finland has been receiving Russian-speaking immigrants, which peaked at the collapse of the Soviet Union in 1991 and subsequently a steady growth. In terms of attitudes toward Russian speakers in Finland, Russian-speaking immigrants are known to be subjected to quite high levels of prejudice and discrimination (see Varjonen et al., 2017). It was previously shown that such experiences prevent people who have immigrated from the former Soviet Union from identifying themselves with the national majority and thus have negative attitudes toward the national majority (Jasinskaja-Lahti et al., 2009).

In Norway, the Russian-speaking immigrant group is considerably smaller compared to Finland and Estonia. As of 2016, when the data for this study were collected, immigrants made up for 13 percent of the total population in Norway, and only 2 percent of the immigrants had Russian as their mother tongue (Statistics Norway, 2016). After the collapse of the Soviet Union, Norway received a large number of immigrants from Russia. Most Russians who migrated to Norway have transnational family ties and intercultural marriages (e.g., Flemmen, 2008; Heyse, 2010; Munkejord, 2017), and the majority of them is first-generation immigrants. While there is not much systematic research done on the integration of Russian-speaking immigrants into Norwegian society, the social status of Russian-speaking immigrants in Norwegian society is not particularly high, perhaps due to possible Russian aggression toward Norway. Thus, Russian-speaking migrants to Norway face some prejudice.

## Materials and Methods

### Participants and Data

The data used in this study were gathered as a part of the international collaborative project called Mutual Intercultural Relations in Plural Societies (MIRIPS), coordinated by Berry (2017). (see *Mutual Intercultural Relations*. Cambridge, Cambridge University Press; and project website: http://www.victoria.ac.nz/cacr/research/mirips). The MIRIPS project investigated various underlying mechanisms of intercultural relations, including but not limited to multiculturalism ideology, contact, acculturation attitudes, and social identification in 17 countries. Estonian and Norwegian data were collected within the MIRIPS sub-project called DIMA, with the help of the Estonian Social and Market Research Company (i.e., Saar Poll). While in Estonia, face-to-face interviews among ethnic Estonian adults were undertaken in 2015, in Norway, a web-based survey was sent to a random sample of native Norwegians using a pre-recruited Computer Assisted Web Interview panel of approximately 58,000 Norwegians. Finnish data were gathered within the MIRIPS-FI project in 2012 through a postal survey among the representative sample of Finnish majority members. Response rates were as follows: 48% for Estonia, 33.5% for Finland, and 36% for Norway. Although the response rates were less than ideal for each country, they were acceptable. In recent years, response rates to surveys are declining in general, mainly due to increased community-based studies and subsequent participation fatigue (e.g., Nulty, 2008; Burgard et al., 2019). Nevertheless, the results of this study should be interpreted cautiously. The overall sample included 1,341 participants (*N* = 506 for Estonia, *N* = 335 for Finland, and *N* = 500 for Norway). The inclusion criteria were the mother tongue, country of birth, and country of residence at the time of the survey. The mean age and gender distribution of the participants per country can be shown in Table 1. The gender and age of the participants were controlled in the analyses while testing the main models.

**Table 1:** Overall and country-specific means and standard deviations of all variables.

	Estonia		Finland		Norway		Overall sample	
	*M*	*SD*	*M*	*SD*	*M*	*SD*	*M*	*SD*
Personal discrimination	1.89	0.90	1.91	0.96	1.85	1.00	1.88	0.95
Group discrimination	3.08	1.03	2.46	1.05	2.32	1.10	2.66	1.11
Personal economic insecurity	2.58	1.24	2.30	1.01	1.97	1.27	2,29	1.22
Group economic insecurity	3.49	0.98	3.26	1.09	2.51	1.00	3.08	1.11
Willingness to confront injustice	2.33	0.75	2.66	0.98	3.62	1.02	2.91	1.09
Age	48.48	16.47	45.87	13.75	51.84	18.37	49.08	16.75
Gender (% female)	53.4		57		46.6		51.8	

### Measures

*Perceived personal ethnic discrimination* from Russian minority group members was measured with two items adapted from the perceived discrimination scale by Berry et al. (2006). The items were “I think that <MINORITY MEMBERS> have something against me because I’m <MAJORITY MEMBER>” and “<MINORITY MEMBERS> have threatened or attacked me because I’m <MAJORITY MEMBER>.” The Spearman-Brown coefficients were 0.65 for the whole data; 0.66 for Estonia, 0.63 for Finland, and 0.68 for Norway.

*Perceived group ethnic discrimination* from Russian minority group members was also measured with two items adapted from Berry et al. (2006) perceived discrimination scale. The items were “In my opinion <MINORITY> have treated <MAJORITY> unfairly or otherwise negatively” and “I think that <MINORITY> do not accept <MAJORITY>.” The Spearman-Brown coefficients were 0.78 for the whole data; 0.76 for Estonia, 0.77 for Finland, and 0.78 for Norway.

*The perceived personal economic insecurity* was assessed with a single question: “What is your or your family’s current economic situation?” The responses ranged from 1 = “We earn/have enough money for our needs and are able to save” to 5 = “We have to cut back on consumption, and we cannot/do not manage on our earnings,” with higher values indicating more personal economic insecurity.

*Perceived group economic insecurity* was assessed with a single item: “Compared to <MAJORITY>, the social and economic standing of <MINORITY> is much worse” and participants’ responses to the item on a 5-point scale that ranged from 1 = “Strongly disagree” to 5 = “Strongly agree.” To achieve compatibility with the corresponding personal measure, the responses were recoded as 1 = “Strongly agree” to 5 = “Strongly disagree,” with higher values indicating higher perceived group economic insecurity.

*Willingness to confront injustice on behalf of the minority* was assessed with two items that tap into the confronting injustice toward minority were adapted from Simon et al. (1998) four-item measure of willingness to participate in (future) collective action on behalf of the minority: “(I would) defend the rights of <MINORITY> in a public debate” and “(I would) intervene verbally in situations in which I notice discrimination of <MINORITY>.” Participants responded to the items on a 4-point scale that ranged from 1 = “No/Totally disagree/ Definitely not ready to” to 4 = “Yes/Totally agree/Definitely ready to” in Finland and Estonia and on a 5-point scale with the same response options as well as an option of “3 = Nor disagree or agree.” To ensure compatibility across the countries, the responses were converted to standardized scores. The Spearman-Brown coefficients were 0.60 for the whole data; 0.60 for Estonia, 0.42 for Finland, and 0.73 for Norway.

*Age* and *gender* were included as covariates in the analyses. Alongside those measures, the survey included other measures which were not used in this study, such as outgroup trust, satisfaction with life, appreciation of Russian culture, intergroup anxiety, and support for multicultural ideology.

### Statistical Analysis

In order to see how different combinations of both personal and group threats predict majorities’ (un)willingness to confront injustice for minority, we employed polynomial regression with subsequent response surface analysis that allows us to see the relationship between the two interrelated predictor variables by producing a three-dimensional figure (Edwards and Parry, 1993; Shanock et al., 2010).

## Results

### Preliminary Analysis

The overall and country-specific descriptive statistics can be shown in Table 1. The results of one-way between groups ANOVA showed that while perceived personal discrimination scores in each country did not differ significantly [*F*(2, 1,269) = 1.45, *p* = 0.236], there was a significant difference among countries in perceived group discrimination [*F*(2, 1,244) = 66.36, *p* < 0.001], perceived personal economic insecurity [*F*(2, 1,315) = 32.66, *p* < 0.001], and perceived group economic insecurity [*F*(2, 1,269) = 115.67, *p* < 0.001]. Post-hoc comparisons showed that the mean of perceived group discrimination in Estonia (*M* = 3.08, *SD* = 1.03) was significantly higher than Norway (*M* = 2.36, *SD* = 1.10) and Finland (*M* = 2.46, *SD* = 1.05), while there were no significant difference between Finland and Norway. Perceived personal and group economic insecurity scores differed significantly among all three countries. Results showed that perceived personal economic insecurity was the highest in Estonia (*M* = 2.58, *SD* = 1.23), followed by Finland (*M* = 2.30, *SD* = 1.01), and lowest in Norway (*M* = 1.97, *SD* = 1.27). Similarly, perceived group economic insecurity was the highest in Estonia (*M* = 3.49, *SD* = 0.98), followed by Finland (*M* = 3.26, *SD* = 1.10), and Norway (*M* = 2.51, *SD* = 1.01). At the level of the whole data, participants perceived higher levels of group threat (both ethnic discrimination and economic insecurity) compared to personal threat, and this difference was significant; *t*(1221) = −33.321, *p* < 0.001 for discrimination and *t*(1252) = 18.483, *p* < 0.001 for economic insecurity. This difference was found in all three countries: *t*(481) = −28.051, *p* < 0.001 for Estonia; *t*(331) = −16.972, *p* < 0.001 for Finland; and *t*(407) = −14.618, *p* < 0.001 for Norway for discrimination, and *t*(485) = 12.709, *p* < 0.001 for Estonia; *t*(326) = 11.756, *p* < 0.001 for Finland; and *t*(439) = 7.980, *p* < 0.001 for Norway for economic insecurity. Table 2 presents the bivariate correlations among the variables for the entire sample. All predictor variables were negatively correlated with willingness to confront injustice. By using the online calculator launched by Lenhard and Lenhard (2014), we found that group threat variables were significantly more strongly correlated with (un)willingness to confront injustice, as compared to the personal threat variables: for ethnic discrimination, *z* = 7.589, *p* < 0.001 and for economic insecurity, *z* = 3.793, *p* < 0.001.

**Table 2:** Pearson correlations among all variables for the combined sample.

S. No.	Variables	1	2	3	4	5	6	7
1.	Personal discrimination	–	0.65^**^	0.10^**^	0.05	−0.27^**^	−0.08^**^	−0.06^*^
2.	Group discrimination		–	0.16^**^	0.10^**^	−0.43^**^	−0.03	0.01
3.	Personal economic insecurity			–	0.13^**^	−0.19^**^	−0.06^*^	0.08^**^
4.	Group economic insecurity				–	−0.32^**^	0.01	0.05
5.	Willingness to confront injustice					–	0.11^**^	−0.06
6.	Age						–	−0.05
7.	Gender (0 = male)							–

* *p* < 0.05;

** *p* < 0.01.

Regarding our main analysis, one of the assumptions of polynomial regression analysis is that there are enough participants in the data who have discrepancies between the two predictors (Shanock et al., 2010). To see if our sample qualifies for this assumption, we followed the procedure introduced by Fleenor et al. (1996) and cited in Shanock et al. (2010). Over half of our sample had discrepant values for both types of predictor variables; only 32.8% of our sample rated their perceptions of personal and group ethnic discrimination to be similar, and 26.5% perceived similar personal and group economic insecurity. Thus, we moved on with the polynomial regression analysis to examine the effect of discrepancies between the different types of threats perceived by the majority groups on the outcome variable.

### Results of the Polynomial Regression and Surface Analysis

By following the procedure of Atwater et al. (2005), we performed a polynomial regression analysis by including the two midpoint centered predictor variables, the square of both these variables, and the cross product of these variables. Instead of evaluating the regression coefficients, we examined four surface test values (Edwards, 2007) by using the Excel macro provided by Shanock et al. (2010). The respective values are as follows: a1 showing the slope along the agreement line (in which both predictors are simultaneously high or low), a2 showing the curvature along the agreement line, a3 showing the direction of the discrepancy along the disagreement line (in which one predictor is high while the other is low, and vice versa), and a4 showing the degree of discrepancy. The results that include the unstandardized coefficients and the surface test values for both models (i.e., Model 1 for perceived ethnic discrimination and Model 2 for perceived economic insecurity) can be shown in Table 3.

**Table 3:** Unstandardized coefficients and the surface test values for Model 1 and 2.

Model 1		Model 2	
Variables	b (SE)	Variables	b (SE)
Constant	−0.47^**^ (0.11)	Constant	−0.61^**^ (0.11)
Age	0.01^**^ (0.01)	Age	0.01^**^ (0.01)
Gender	−0.11 (0.06)	Gender	−0.06 (0.06)
Perceived personal discrimination (PPD)	0.08 (0.08)	Perceived personal economy (PPE)	−0.06^*^ (0.03)
Perceived group discrimination (PGD)	−0.40^**^ (0.07)	Perceived group economy (PGE)	−0.27^**^ (0.03)
PPD^2^	0.04 (0.04)	PPE^2^	0.08^**^ (0.02)
PPD × PGD	−0.01 (0.06)	PPE × PGE	0.02 (0.02)
PGD^2^	0.04 (0.03)	PGE^2^	0.01 (0.02)
*R*^2^/Δ*R*^2^	0.20/0.19^**^	*R*^2^/Δ*R*^2^	0.15/0.15^**^
Surface tests		Surface tests	
Agreement line		Agreement line	
Slope (a1)	−0.33^**^	Slope (a1)	−0.34^**^
Curvature (a2)	0.07	Curvature (a2)	0.12^**^
Disagreement line		Disagreement line	
Slope (a3)	0.48^**^	Slope (a3)	0.21^**^
Curvature (a4)	0.09	Curvature (a4)	0.08

* *p* < 0.05;

** *p* < 0.01.

#### Model 1 – Perceived Personal and Group Ethnic Discrimination

**Figure 1:**
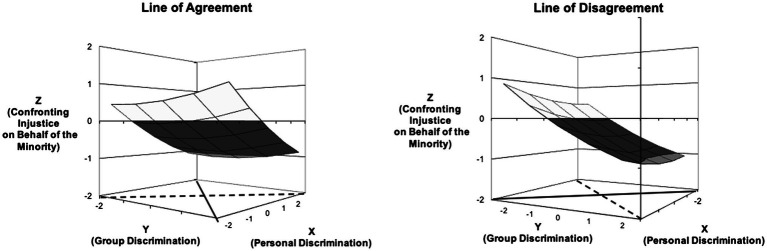
Three-dimensional visual representation of the surface values for the Model 1.

Figure 1 shows the three-dimensional response surface based on the surface test values. The line of agreement is depicted with a dashed line, and the line of disagreement is depicted with a straight line. The results show that perceived personal and group discrimination jointly predict (un) willingness to confront injustice along the agreement line, and the relationship is a negative and linear one (slope test statistic = −0.33, *p* < 0.001). This indicates that those national majority group members who perceived higher levels of both personal and group discrimination were more unwilling to confront injustice on behalf of the Russian minority (see the figure on the left side).

The two types of perceived ethnic discrimination also jointly predicted unwillingness to confront injustice along the disagreement line (slope test statistic = 0.48, *p* < 0.001). A significant positive slope shows the direction of the discrepancy: Unwillingness to confront injustice for the Russian minority was higher when the lower levels of perceived personal discrimination were accompanied by the higher levels of perceived group discrimination (see the figure on the right side). Thus, both of our hypotheses (H1a and H2a) for the association between personal- and group perceived discrimination and unwillingness to confront injustice faced by the Russian minority were supported.

#### Model 2 – Perceived Personal and Group Economic Insecurity

**Figure 2:**
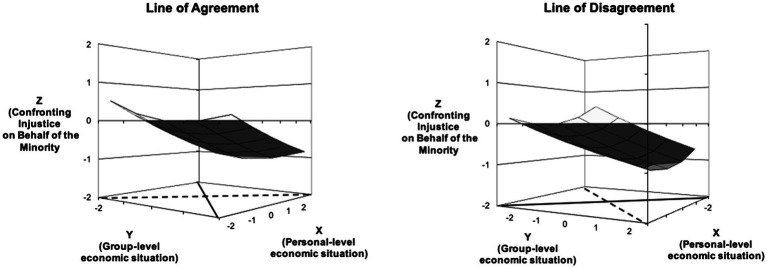
Three-dimensional visual representation of the surface values for the Model 2.

Figure 2 is the graph that shows the response surface test results for Model 2. Again, the agreement between the perceived personal and group economic insecurity significantly predicted the unwillingness of the majority to confront injustice on behalf of the minority. The linear slope was again significant and negative (slope test statistic = −0.34, *p* < 0.001), suggesting that majority group members who perceived heightened economic insecurity both personally and at the group level were more unwilling to confront injustice for the Russian minority. The curvature test statistics along the agreement line were also significant and positive (a2 = 0.12, *p* < 0.01), pointing toward a slight upward curve along the agreement line. The curve was especially visible toward the unwillingness (or right corner of the figure) – the sharp decline in willingness to confront injustice stops and turns to a straighter line after the midpoint of the scale. This suggests that unwillingness to confront injustice increases significantly with both personal and group perceptions of economic situation changing from perceived economic security into average standing.

Similar to Model 1, the relationship between the two types of perceived economic insecurity and willingness to confront injustice was also significant along the line of disagreement – unwillingness to confront injustice was higher among those who perceived heightened group, but less personal economic insecurity (slope test statistic = 0.21, *p* < 0.001). The curvature along the line of disagreement was also marginally significant (curvature test statistic = 0.08, *p* = 0.056), pointing to a convex surface and suggesting that the willingness to confront injustice decreases toward the midpoint of both scales. In other words, those who perceived their personal economic insecurity and their country’s economic insecurity as similar, and those who perceived more group but less personal economic insecurity were similarly reluctant to confront injustice (see the figure on the right side). Again, both of our hypotheses H1b and H2b were supported.

### Country-Specific Results

As the next step, we explored country-specific results for either of the two models. We re-ran the polynomial regression analysis for all three countries separately. Model 1, in which perceived personal and group discrimination were the predictors, both predictors were significant in all the three countries; Estonia, *R*^2^ = 0.11, *F*(7, 434) = 7.70, *p* < 0.001; Finland, *R*^2^ = 0.32, *F*(7,196) = 13.17, *p* < 0.001; and Norway, *R*^2^ = 0.13, *F*(7,366) = 7.93, *p* < 0.001. However, some differences were observed when it came to the country-specific response surface analysis. In Estonia, the agreement between perceived personal and group ethnic discrimination significantly predicted unwillingness to confront injustice linearly (slope test statistic = −0.31, *p* < 0.001). The curvature test statistic was also significant (−0.06, *p* = 0.01), suggesting that the Estonians who perceived moderate to no personal and group discrimination were equally willing to confront injustice. While the disagreement between the two discrimination types did not significantly predict the outcome, the direction of the relationship was the same as in the overall model (slope test statistic = 0.21, *p* = 0.192; curvature test statistic = 0.11, *p* = 0.313). In Finland, in line with the overall model, the agreement between perceived personal and group discrimination significantly and linearly predicted the outcome. Still, the relationship was a negative one (slope test statistic = −0.51, *p* < 0.001). However, the disagreement between the two types of discrimination did not significantly predict unwillingness to confront injustice (slope test statistic = 0.04, *p* = 0.911). Finally, in Norway, the two predictors significantly predicted the outcome along the agreement line only curvilinearly (curvature test statistic = 0.15, *p* = 0.001), suggesting that those Norwegians who perceived either high or low level of both types of discrimination were less unwilling to confront injustice compared to those who perceive moderate levels of both types of discrimination. The relationship along the disagreement line did not significantly predict the outcome (slope test statistic = 0.34, *p* = 0.096).

Model 2, in which perceived personal and group economic insecurities were the predictors, was significant for Finland, *R*^2^ = 0.13, *F*(7,191) = 4.19, *p* < 0.001 and for Norway, *R*^2^ = 0.04, *F*(7,394) = 2.36, *p* = 0.01 but not for Estonia, *R*^2^ = 0.02, *F*(7,437) = 1.27, *p* = 0.27. In Finland, the two predictors were not associated with unwillingness to confront injustice along the agreement line. Like in the overall model, they significantly and positively predicted the outcome along the disagreement line (slope test statistic = 0.37, *p* = 0.001). In Norway, all of the test statistics were in the same direction as the significant tests of the overall model, although none of them reached significance.

To conclude, we obtained evidence that there are simultaneous effects of perceived personal and group realistic threats in terms of ethnic discrimination (Model 1) and economic insecurity (Model 2) on majorities’ unwillingness to confront injustice. Notably, the results showed a cumulative effect of majority members’ perceptions of personal and group threats on their unwillingness to support minority groups. Although some of the slope tests did not reach statistical significance, the directions of almost all the associations in the country-specific models generally aligned with the associations found in the overall model. The exploration of between-country differences produced two notions about Norway and Estonia, which we will discuss in the concluding section.

## Discussion

This study sought to uncover threat-related mechanisms that may underlie advantaged majority group members’ unwillingness to confront injustice on behalf of disadvantaged minority groups of Russian speakers in Estonia, Finland, and Norway. In line with Stephan and Renfro’s (2002) arguments, we distinguished between personal and group threats (Taylor et al., 1994; Postmes et al., 1999). We argued that perceived personal and group realistic threats, namely ethnic discrimination and economic insecurity, could explain national majority members’ unwillingness to confront injustice on behalf of the minority group (see Stephan and Stephan, 2000).

### Strengths of the Current Study

Our results showed that perceptions of high levels of ethnic discrimination in all three countries studied and perceptions of economic insecurity in Finland and Norway predicted more unwillingness to confront injustice on behalf of the Russian minority when these threats accumulated (i.e., were perceived both personally and on behalf of the in-group). Thus, our first hypothesis was confirmed, supporting previous research showing how cumulative, multifaceted realistic threats may exacerbate negative intergroup outcomes (see DiPrete and Eirich, 2006; Renvik et al., 2018). Previous research often linked discrimination experiences with outgroup negativity (e.g., Jasinskaja-Lahti et al., 2018) and with the willingness to engage in various forms of collective action to support and promote the rights of the in-group (SIMCA; van Zomeren et al., 2011) among minority groups. Our findings suggest that perceived ethnic discrimination – be it targeted to an individual personally or toward one’s in-group – may also hinder majority group members’ willingness to promote equal rights of disadvantaged groups. Furthermore, perceptions of not being economically better-off than Russian-speaking minority group members (Runciman, 1966; Jetten et al., 2015) also predicted the unwillingness of national majority group members to confront injustice for the minority group. Indeed, perceptions of the outgroup doing economically better as compared to the standing of the national majority may motivate the advantaged group members to withdraw their support from any actions that would make a change to the current social order in which the advantaged group has a higher status and more power (Jetten, 2019; Li, 2019).

With our methodological approach, we were also able to identify patterns of relationship between perceived personal and group threats that specifically predicted the majority’s unwillingness to confront injustice on behalf of the minority. While both perceived personal and group threats were jointly associated with unwillingness to confront injustice, for both ethnic discrimination and economic insecurity, threats toward the in-group were important predictors of unwillingness to confront injustice on behalf of the minority group. When the in-group was perceived to be doing fine, perceived personal threats did not prevent the majority group from supporting the minority Russian-speaking immigrants. In reverse, the perception of being personally advantaged and perceiving one’s in-group to be in a worse situation predicted reluctance to stand up against social inequality faced by minority Russian-speakers. These findings are also in line with previous research that showed that group disadvantage is perceived to a higher degree than personal disadvantage (e.g., Taylor et al., 1990) and that when social identities and intergroup tensions are made salient, people react on an intergroup level (Tajfel and Turner, 1979; see also Guimond and Dubé-Simard, 1983; Pettigrew and Meertens, 1995; Pettigrew et al., 2008; Sassenberg and Woltin 2009).

Furthermore, previous research on relative gratification suggested that feelings of being better-off than the outgroup may increase negativity and hostility toward the outgroup due to the perception of threat and fear of future loss (Guimond and Dambrun, 2002; Jetten et al., 2015). Our findings, however, are in line with SIT (Tajfel and Turner, 1979): It is not only the lack of perceived personal and group ethnic discrimination but also perceived economic security of the individual and the in-group that were linked to more readiness to confront injustice. When majority group members feel that their own and their in-group’s status are safe, they “can afford” to supporting the minority’s actions to promote their in-group’s status (e.g., Turner and Reynolds, 2001; Jetten, 2019). Another explanation of these results might be that perceiving to be wealthier than the disadvantaged group may make advantaged group members realize and condemn this violation of the moral convictions against social inequality (van Zomeren et al., 2011).

It is important to recognize that while we were primarily interested in general social-psychological processes and concerned with national majorities’ willingness to confront injustice toward ethnic minorities, the status and the history of Russian-speakers vary in the three countries studied. Nevertheless, our country-specific results showed that the statistically significant surface test values were in the same direction as in the overall model. Of the significant models, the most considerable variation was found in Norway. Norwegians who perceived moderate levels of both types of discrimination were the ones who were most reluctant to confront injustice for Russian immigrants in Norway. This reluctance may be rooted in the strong Norwegian cultural value of maintaining “peace and quiet” around them (Gullestad, 1992). Norwegians tend to avoid conflictual interactions when differences are perceived as big. In other words, Norwegians with moderate perceptions of ethnic discrimination see no reason to act and possibly increase intergroup conflict but to let things remain as they are. It should also be noted that the Russian-speaking immigrant community in Norway is not very sizeable compared with the sizes in the two other countries, Estonia and Finland. Combined with the economic prosperity of Norway, these differences are probably reflected in the lower levels of perceived threats (both personal and group based), a weaker association between perceived intergroup threats, and negativity toward this minority group in the Norwegian context. Another interesting difference was found in Estonia, where cumulative economic insecurity did not predict reactions toward the Russian-speaking minority. We assume that this is due to the considerable size of the established Russian-speaking minority and their challenges with cultural integration to the society (see, e.g., Kruusvall et al., 2009). It is possible that more cultural than economic insecurities drive the reactions of the national majority group members toward the Russian-speaking minority in Estonia.

Related to the latter point, in future research, it would be fruitful to include perceived symbolic threats, and this may be a better reflection of problems in the area of cultural integration. Further, we recognize that while perceived economic insecurity and perceived discrimination can be regarded as threats to the in-group’s positive distinctiveness and value, respectively (see Branscombe et al., 1999), they are only two forms of social identity threats. Future studies would benefit from focusing on majority groups’ solidarity toward minorities together with other types of perceived threats. Finally, while our study focused mainly on perceived threats and *unwillingness* to help the minority outgroup, it would be interesting to focus also on more positive mechanisms behind *willingness* or readiness to help, such as those related to intergroup empathy (see, e.g., Vanman, 2016).

### Limitations

Despite the clarity of our findings, the limitations of the study cannot be overlooked. Firstly, all four predictors were either assessed with single items or with two-item measures, which are not optimal on psychometric grounds. It is also important to point out that while the personal economic insecurity item was a question that directly assessed the current economic situation of the participant, group economic insecurity was assessed with an item that includes a clear comparison between the in-group (majority) and the outgroup (Russian-speaking minority). While it would have been optimal to have matched the personal and group items, the two items separately measured perceptions of personal and group economic insecurity, our approach fits very well with our overall research aims. Lastly, it should be noted that the design of the study is cross-sectional, and any notion of causal association should be done cautiously. Our assumptions and hypotheses were drawn from both classical theories, and several studies that have found a causal link between feelings of disadvantage and collective action.

It is also important to justify why we limited our attention to willingness to confront injustice and ignored participation in other forms of collective action, such as participation in social protests and demonstrations. The concept of “speaking out” when confronting unfairness has been previously acknowledged in the study of behavioral intentions by Louis et al. (2007). The two items that were adapted from the willingness to participate in the collective action scale of Simon et al. (1998) were previously used as a subscale of willingness to confront injustice by Jasinskaja-Lahti et al. (2018). We proceeded with using the adapted two-item confronting injustice scale due to Russian immigrants/minorities not being a specific, clear-cut group with determined political agendas in Nordic countries and Estonia. More active forms of collective action, such as signing petitions and protest participation, are not very common among them (Jasinskaja-Lahti et al., 2018). Still, Russian immigrants and minorities face high levels of discrimination in these countries, and not only themselves but also all members of the larger society need to be supportive of and engaged in publicly confronting injustice toward others. Thus, for having a contextually appropriate scale, we chose to use the two items that we believe tap into the willingness to confront injustice on behalf of the disadvantaged.

## Conclusion

Perhaps, the most significant contribution of this study is the recognition of some critical underlying mechanisms (i.e., perceived ethnic discrimination and economic insecurity) that can lead to advantaged group members’ unwillingness to confront injustice on behalf of the minority. Furthermore, our results have some theoretical implications by showing the prominent role of group evaluations as a predictor of disadvantaged minority group support and also the importance of focusing on possible non-linear associations between personal and group threats on the one hand and collective action intentions on the other hand. Additionally, we found that perceptions of heightened personal and group realistic threats cumulatively led to more unwillingness to confront injustice. Our study emphasized the importance of recognizing both personal and group evaluations and how group threats play a crucial role in the unwillingness to confront injustice on behalf of the disadvantaged. With our results, we point out that to move toward a more equal society, we need to better understand the psychological obstacles that make national majorities more unwilling to strive for social equality. The task is crucial, as these groups have much power over minorities’ integration and social cohesion in society.

## Data Availability Statement

The datasets presented in this study can be found in online repositories. The names of the repository/repositories and accession number(s) can be found at: https://osf.io/p7d96/.

## Ethics Statement

All procedures performed in studies involving human participants were in accordance with the ethical standards of the Academy of Finland, and with the 1964 Helsinki Declaration and its later amendments or comparable ethical standards. The patients/participants provided their written informed consent to participate in this study.

## Author Contributions

GC has produced the initial and revised drafts of the manuscript, has led the writing and revision processes, and conducted the main statistical analysis. IJ-L and TR have made substantial contributions to the theoretical introduction and the discussion of the manuscript. GC, IJ-L, and TR contributed to the theoretical concept, design of the study, the interpretation of the data. RV and DS have contributed to collecting the data and commenting on the final version of the manuscript as they saw appropriate. All authors contributed to the article and approved the submitted version.

### Conflict of Interest

The authors declare that the research was conducted in the absence of any commercial or financial relationships that could be construed as a potential conflict of interest.

## References

[ref1] Atwater L. Waldman D. Ostroff C. Robie C. Johnson K. M. (2005). Self–other agreement: comparing its relationship with performance in the U.S. and Europe. Int. J. Sel. Assess. 13, 25–40. 10.1111/j.0965-075X.2005.00297.x

[ref006] Aure M. (2011). Borders of Understanding: Re-making Frontiers in the Russian–Norwegian Contact Zone. Ethnopolitics. Ethnopolitics. 10:2, 171–186. 10.1080/17449057.2011.570981

[ref2] Bagci S. C. Çelebi E. (2017). Cross-group friendships and outgroup attitudes among Turkish-Kurdish ethnic groups: does perceived interethnic conflict moderate the friendship-attitude link? J. Appl. Soc. Psychol. 47, 59–73. 10.1111/jasp.12413

[ref3] Barrington L. (1995). Nations, states, and citizens: an explanation of the citizenship policies in Estonia and Lithuania. Rev. Cent. East Eur. Law 21, 103–148. 10.1163/157303595X00066

[ref4] Bergmann B. R. (1999). The continuing need for affirmative action. Q. Rev. Econ. Finance 39, 757–768. 10.1016/S1062-9769(99)00027-7

[ref5] Berry J. W. (ed.) (2017). Mutual Intercultural Relations. Cambridge: Cambridge University Press.

[ref6] Berry J. W. Phinney J. S. Sam D. L. Vedder P. (eds.) (2006). Immigrant Youth in Cultural Transition: Acculturation, Identity, and Adaptation Across National Contexts. Mahwah, NJ, USA: Lawrence Erlbaum.

[ref7] Boeri T. Hanson G. McCormick B. (eds.) (2002). Immigration Policy and the Welfare System. Oxford: Oxford University Press.

[ref8] Branscombe N. R. Ellemers N. Spears R. Doosje B. (1999). “The context and content of social identity threat,” in Social Identity: Context, Commitment, Content. eds. Ellemers N. Spears R. Doosje B. (New Jersey: Wiley-Blackwell), 35–58.

[ref11] Brysk A. Wehrenfennig D. (2010). ‘My brother's keeper’? Inter-ethnic solidarity and human rights. Stud. Ethn. Natl. 10, 1–18. 10.1111/j.1754-9469.2010.01067.x

[ref009] Burgard T. Kasten N. Bosnjak M. (2019). Participation in online surveys in psychology. A meta-analysis. ZPID (Leibniz Institute for Psychology Information). 10.23668/psycharchives.2473

[ref12] Burhan O. K. van Leeuwen E. (2016). Altering perceived cultural and economic threats can increase immigrant helping. J. Soc. Issues 72, 548–565. 10.1111/josi.12181

[ref15] Celikkol G. Mähönen T. A. Jasinskaja-Lahti I. (2017). The interplay between objective and subjective ethnocultural diversity in predicting intergroup relations. J. Ethn. Migr. Stud. 43, 1399–1416. 10.1080/1369183X.2016.1238758

[ref16] Cottrell C. A. Neuberg S. L. (2005). Different emotional reactions to different groups: a sociofunctional threat-based approach to “prejudice”. J. Pers. Soc. Psychol. 88, 770–789. 10.1037/0022-3514.88.5.770, PMID: 15898874

[ref17] Crosby F. (1984). The denial of personal discrimination. Am. Behav. Sci. 27, 371–386. 10.1177/000276484027003008

[ref18] Dambrun M. Taylor D. M. McDonald D. A. Crush J. Méot A. (2006). The relative deprivation gratification continuum and the attitudes of South Africans toward immigrants: a test of the V-curve hypothesis. J. Pers. Soc. Psychol. 91, 1032–1044. 10.1037/0022-3514.91.6.1032, PMID: 17144763

[ref19] Denissen J. J. Bleidorn W. Hennecke M. Luhmann M. Orth U. Specht J.. (2018). Uncovering the power of personality to shape income. Psychol. Sci. 29, 3–13. 10.1177/0956797617724435, PMID: 29155616 PMC5774615

[ref20] DiPrete T. A. Eirich G. M. (2006). Cumulative advantage as a mechanism for inequality: a review of theoretical and empirical developments. Annu. Rev. Sociol. 32, 271–297. 10.1146/annurev.soc.32.061604.123127

[ref21] Dover T. L. Major B. Kaiser C. R. (2016). Members of high-status groups are threatened by pro-diversity organizational messages. J. Exp. Soc. Psychol. 62, 58–67. 10.1016/j.jesp.2015.10.006

[ref22] Dube L. Guimond S. (1986). “Relative deprivation and social protest: the personal-group issue,” in Relative Deprivation and Social Comparison: The Ontario Symposium. *Vol*. 4. eds. Olson M. Herman C. P. Zanna M. P. (Hillsdale, NJ: Erlbaum), 201–216.

[ref002] Duckitt J. (2001). A dual-process cognitive-motivational theory of ideology and prejudice. Advances in experimental social psychology. 33, 41–113. 10.1016/S0065-2601(01)80004-6

[ref23] Edwards J. R. (2007). “Polynomial regression and response surface methodology,” in Perspectives on Organizational Fit. eds. Ostroff C. Judge T. A. (San Francisco: Jossey-Bass), 361–372.

[ref24] Edwards J. R. Parry M. E. (1993). On the use of polynomial regression equations as an alternative to difference scores in organizational research. Acad. Manag. J. 36, 1577–1613. 10.5465/256822

[ref25] Esses V. M. Jackson L. M. Armstrong T. L. (1998). Intergroup competition and attitudes toward immigrants and immigration: an instrumental model of group conflict. J. Soc. Issues 54, 699–724. 10.1111/j.1540-4560.1998.tb01244.x

[ref26] Ferwerda J. Flynn D. J. Horiuchi Y. (2017). Explaining opposition to refugee resettlement: the role of NIMBYism and perceived threats. Sci. Adv. 3:e1700812. 10.1126/sciadv.1700812, PMID: 28913425 PMC5587019

[ref27] Fleenor J. W. McCauley C. D. Brutus S. (1996). Self-other rating agreement and leader effectiveness. Leadersh. Q. 7, 487–506. 10.1016/S1048-9843(96)90003-X

[ref28] Flemmen A. B. (2008). Transnational marriages–empirical complexities and theoretical challenges. An exploration of intersectionality. Nordic J. Femin. Gen. Res. 16, 114–129. 10.1080/08038740802140244

[ref29] Grigoryev D. Batkhina A. van de Vijver F. Berry J. W. (2020). Towards an integration of models of discrimination of immigrants: from ultimate (functional) to proximate (sociofunctional) explanations. J. Int. Migr. Integr. 21, 667–691. 10.1007/s12134-019-00677-w

[ref30] Grofman B. N. Muller E. N. (1973). The strange case of relative gratification and potential for political violence: the V-curve hypothesis. Am. Polit. Sci. Rev. 67, 514–539. 10.2307/1958781

[ref31] Guimond S. Dambrun M. (2002). When prosperity breeds intergroup hostility: the effects of relative deprivation and relative gratification on prejudice. Personal. Soc. Psychol. Bull. 28, 900–912. 10.1177/014616720202800704

[ref32] Guimond S. Dubé-Simard L. (1983). Relative deprivation theory and the Quebec nationalist movement: the cognition–emotion distinction and the personal–group deprivation issue. J. Pers. Soc. Psychol. 44, 526–535. 10.1037/0022-3514.44.3.526

[ref33] Gullestad M. (1992). The Art of Social Relations: Essays on Culture, Social Action and Everyday Life in Modern Norway. Oxford: Oxford University Press.

[ref34] Hasbún López P. Martinović B. Bobowik M. Chryssochoou X. Cichocka A. Ernst-Vintila A.. (2019). Support for collective action against refugees: the role of national, European, and global identifications, and autochthony beliefs. Eur. J. Soc. Psychol. 49, 1439–1455. 10.1002/ejsp.2608, PMID: 31894165 PMC6919941

[ref35] Heyse P. (2010). Deconstructing fixed identities: an intersectional analysis of Russian speaking female marriage migrants’ self-representations. J. Intercult. Stud. 31, 65–80. 10.1080/07256860903487661

[ref36] Inglehart R. F. Norris P. (2016). Trump, Brexit, and the rise of populism: economic have-nots and cultural backlash. Harvard Kennedy School Faculty Research Working Paper Series RWP16-026.

[ref37] Jackson L. M. Esses V. M. (2000). Effects of perceived economic competition on people’s willingness to help empower immigrants. Group Process. Intergroup Relat. 3, 419–435. 10.1177/1368430200003004006

[ref001] Jaakkola M. (2000). “Finnish attitudes towards immigrants in 1987-1999,”. Finnish Yearbook of Population Research. 36, 129–161. 10.23979/fypr.44951

[ref38] Jasinskaja-Lahti I. Celikkol G. Renvik T. A. Eskelinen V. E. Vetik R. Sam D. (2018). When psychological contract is violated: revisiting the rejection-disidentification model of immigrant integration. J. Soc. Polit. Psychol. 6, 484–510. 10.5964/jspp.v6i2.890

[ref39] Jasinskaja-Lahti I. Liebkind K. Solheim E. (2009). To identify or not to identify? National dis-identification as an alternative reaction to perceived ethnic discrimination. Appl. Psychol. 58, 105–128. 10.1111/j.1464-0597.2008.00384.x

[ref40] Jetten J. (2019). The wealth paradox: prosperity and opposition to immigration. Eur. J. Soc. Psychol. 49, 1097–1113. 10.1002/ejsp.2552

[ref41] Jetten J. Mols F. Postmes T. (2015). Relative deprivation and relative wealth enhances anti-immigrant sentiments: the v curve re-examined. PLoS One 10:e0139156. 10.1371/journal.pone.0139156, PMID: 26461497 PMC4604204

[ref43] Kluegel J. Bobo L. (2001). “Perceived group discrimination and policy attitudes: the sources and consequences of the race and gender gaps,” in Urban Inequality: Evidence From Four Cities. eds. O’Connor A. Tilly C. Bobo L. D. (New York: Russell Sage Foundation), 163–216.

[ref44] Kotzur P. F. Schäfer S. J. Wagner U. (2019). Meeting a nice asylum seeker: intergroup contact changes stereotype content perceptions and associated emotional prejudices and encourages solidarity-based collective action intentions. Br. J. Soc. Psychol. 58, 668–690. 10.1111/bjso.12304 30512181

[ref45] Kruusvall J. Vetik R. Berry J. W. (2009). The strategies of inter-ethnic adaptation of Estonian Russians. Stud. Trans. States Soc. 1, 3–24. Available at: https://nbn-resolving.org/urn:nbn:de:0168-ssoar-364001

[ref007] Lenhard W. Lenhard A. (2014). Hypothesis Tests for Comparing Correlations Available at: https://www.psychometrica.de/correlation.html. Bibergau (Germany): Psychometrica.

[ref46] LeVine R. A. Campbell D. T. (1972). Ethnocentrism: Theories of Conflict, Ethnic Attitudes, and Group Behavior. New York: Wiley.

[ref47] Li M. (2019). Priming mediated vicarious intergroup contact: how narrative focus influences attitude changes toward gay people, same-sex family, and social dominance. Imagin. Cogn. Pers. 39, 151–174. 10.1177/0276236618810203

[ref005] Louis W. R. Duck J. M. Terry D. J. Schuller R. A. Lalonde R. N. (2007). Why do citizens want to keep refugees out? Threats, fairness and hostile norms in the treatment of asylum seekers. Eur. J. Soc. Psychol. 37, 53–73. 10.1002/ejsp.329

[ref48] Mallett R. Huntsinger J. R. Sinclair S. Swim J. (2008). Seeing through their eyes: when majority group members take collective action on behalf of an outgroup. Group Process. Intergroup Relat. 11, 451–470. 10.1177/1368430208095400

[ref49] Mayda A. (2006). Who is against immigration? A cross-country investigation of individual attitudes toward immigrants. Rev. Econ. Stat. 88, 510–530. 10.1162/rest.88.3.510

[ref50] Mols F. Jetten J. (2017). The Wealth Paradox. Economic Prosperity and the Hardening of Attitudes. Cambridge, UK: Cambridge University Press.

[ref51] Munkejord M. C. (2017). Local and transnational networking among female immigrant entrepreneurs in peripheral rural contexts: perspectives on Russians in Finnmark, Norway. Eur. Urban Reg. Stud. 24, 7–20. 10.1177/0969776415587122

[ref52] Norton M. I. Sommers S. R. (2011). Whites see racism as a zero-sum game that they are now losing. Perspect. Psychol. Sci. 6, 215–218. 10.1177/1745691611406922, PMID: 26168512

[ref003] Nulty D. D. (2008). The adequacy of response rates to online and paper surveys: what can be done?. Assessment and Evaluation in Higher Education. 33, 301–314. 10.1080/02602930701293231

[ref54] Pettigrew T. F. Christ O. Wagner U. Meertens R. W. Van Dick R. Zick A. (2008). Relative deprivation and intergroup prejudice. J. Soc. Issues 64, 385–401. 10.1111/j.1540-4560.2008.00567.x

[ref55] Pettigrew T. F. Meertens R. W. (1995). Subtle and blatant prejudice in Western Europe. Eur. J. Soc. Psychol. 25, 57–75. 10.1002/ejsp.2420250106

[ref56] Postmes T. Branscombe N. R. Spears R. Young H. (1999). Comparative processes in personal and group judgments: resolving the discrepancy. J. Pers. Soc. Psychol. 76, 320–338. 10.1037/0022-3514.76.2.320

[ref57] Quinn K. A. Roese N. J. Pennington G. L. Olson J. M. (1999). The personal/group discrimination discrepancy: the role of informational complexity. Personal. Soc. Psychol. Bull. 25, 1430–1440. 10.1177/0146167299259008

[ref58] Reimer N. K. Becker J. C. Benz A. Christ O. Dhont K. Klocke U.. (2017). Intergroup contact and social change: implications of negative and positive contact for collective action in advantaged and disadvantaged groups. Personal. Soc. Psychol. Bull. 43, 121–136. 10.1177/0146167216676478, PMID: 28903647

[ref59] Renvik T. A. Brylka A. Konttinen H. Vetik R. Jasinskaja-Lahti I. (2018). Perceived status and national belonging: the case of Russian speakers in Finland and Estonia. Int. Rev. Soc. Psychol. 31, 1–10. 10.5334/irsp.149

[ref61] Runciman W. G. (1966). Relative Deprivation and Social Justice: A Study of Attitudes to Social Inequality in Twentieth-Century England. Berkeley: University of California Press.

[ref004] Sassenberg K. Woltin K.-A. (2009). A self-regulation approach to group processes. in Intergroup relations: The role of motivation and emotion. eds. Otten S. Sassenberg K. Kessler T.. Psychology Press, 101–120.

[ref62] Schmuck D. Matthes J. (2014). How anti-immigrant right-wing populist advertisements affect young voters: symbolic threats, economic threats and the moderating role of education. J. Ethn. Migr. Stud. 41, 1577–1599. 10.1080/1369183X.2014.981513

[ref64] Shanock L. R. Baran B. E. Gentry W. A. Pattison S. C. Heggestad E. D. (2010). Polynomial regression with response surface analysis: a powerful approach for examining moderation and overcoming limitations of difference scores. J. Bus. Psychol. 25, 543–554. 10.1007/s10869-010-9183-4

[ref65] Shepherd L. Fasoli F. Pereira A. Branscombe N. R. (2018). The role of threat, emotions, and prejudice in promoting collective action against immigrant groups. Eur. J. Soc. Psychol. 48, 447–459. 10.1002/ejsp.2346

[ref66] Sherif M. (1966). In Common Predicament: Social Psychology of Intergroup Conflict and Cooperation. Boston: Houghton-Mifflin.

[ref67] Sidanius J. Pratto F. (1999). Social Dominance: An Intergroup Theory of Social Hierarchy and Oppression. New York: Cambridge University Press.

[ref68] Simon B. Loewy M. Stürmer S. Weber U. Freytag P. Habig C.. (1998). Collective identification and social movement participation. J. Pers. Soc. Psychol. 74, 646–658. 10.1037/0022-3514.74.3.646

[ref008] Sirin C. V. Villalobos J. D. Valentino N. A. (2016). Group Empathy Theory: The effect of group empathy on US intergroup attitudes and behavior in the context of immigration threats. J. Polit. 78, 893–908. 10.1086/685735

[ref69] Smith H. J. Pettigrew T. F. Pippin G. M. Bialosiewicz S. (2012). Relative deprivation: a theoretical and meta-analytic review. Personal. Soc. Psychol. Rev. 16, 203–232. 10.1177/1088868311430825, PMID: 22194251

[ref70] Smith H. J. Spears R. (1996). Ability and outcome evaluations as a function of personal and collective (dis)advantage: a group escape from individual bias. Personal. Soc. Psychol. Bull. 22, 635–642. 10.1177/0146167296226008

[ref71] Spruyt B. Keppens G. Van Droogenbroeck F. (2016). Who supports populism and what attracts people to it? Polit. Res. Q. 69, 335–346. 10.1177/1065912916639138

[ref72] Statistics Finland (2019). Population structure. Available at: https://www.tilastokeskus.fi/tup/suoluk/suoluk_vaesto_en.html (Accessed April 1, 2021).

[ref73] Statistics Norway (2016). Immigrants and Norwegian-born to immigrant parents. Available at: https://www.ssb.no/en/befolkning/statistikker/innvbef/aar/2016-03-03 (Accessed January 1, 2016).

[ref74] Stefaniak A. Mallett R. K. Wohl M. J. A. (2020). Zero-sum beliefs shape advantaged allies’ support for collective action. Eur. J. Soc. Psychol. 50, 1259–1275. 10.1002/ejsp.2674

[ref75] Stephan W. G. Renfro C. L. (2002). “The role of threat in intergroup relations,” in From Prejudice to Intergroup Emotions: Differentiated Reactions to Social Groups. eds. Mackie D. M. Smith E. R. (New York: Psychology Press), 191–207.

[ref76] Stephan W. G. Stephan C. W. (2000). “An integrated threat theory of prejudice,” in Reducing Prejudice and Discrimination. ed. Oskamp S. (Mahwah, NJ: Erlbaum), 23–45.

[ref77] Subašić E. Reynolds K. J. Turner J. C. (2008). The political solidarity model of social change: dynamics of self-categorization in intergroup power relations. Personal. Soc. Psychol. Rev. 12, 330–352. 10.1177/1088868308323223, PMID: 18927471

[ref78] Tajfel H. Turner J. (1979). “An integrative theory of intergroup conflict,” in The Social Psychology of Intergroup Relations. eds. Austin W. Worchel S. (Monterey, CA: Brooks/Cole), 33–47.

[ref79] Taylor D. M. Wright S. C. Moghaddam F. M. Lalonde R. N. (1990). The personal/group discrimination discrepancy: perceiving my group but not myself to be a target for discrimination. Personal. Soc. Psychol. Bull. 16, 256–262. 10.1177/0146167290162006

[ref80] Taylor D. M. Wright S. C. Porter L. E. (1994). “Dimensions of perceived discrimination: the personal/group discrimination discrepancy,” in The Psychology of Prejudice: The Ontario Symposium. *Vol*. 7. eds. Zanna M. P. Olson J. M. (Hillsdale, NJ: Lawrence Erlbaum Associates, Inc.), 233–255.

[ref82] Turner J. C. Reynolds K. J. (2001). “The social identity perspective in intergroup relations: theories, themes, and controversies,” in Blackwell Handbook of Social Psychology: Intergroup Processes. eds. Brown R. Gaertner S. L. (Blackwell), 133–152.

[ref83] van Zomeren M. Postmes T. Spears R. (2008). Toward an integrative social identity model of collective action: a quantitative research synthesis of three socio-psychological perspectives. Psychol. Bull. 134, 504–535. 10.1037/0033-2909.134.4.504, PMID: 18605818

[ref84] van Zomeren M. Postmes T. Spears R. Bettanche K. (2011). Can moral convictions motivate the advantaged to challenge social inequality? Extending the social identity model of collective action. Group Process. Intergroup Relat. 14, 735–753. 10.1177/1368430210395637

[ref85] Vanman E. J. (2016). The role of empathy in intergroup relations. Curr. Opin. Psychol. 11, 59–63. 10.1016/j.copsyc.2016.06.007

[ref86] Vanneman R. D. Pettigrew T. F. (1972). Race and relative deprivation in the urban United States. Race 13, 461–486. 10.1177/030639687201300404

[ref87] Varjonen S. Zamiatis A. Rinas M. (2017). Suomen Venäjänkieliset: Tässä ja Nyt. Tilastot, Tutkimukset, Järjestökentän Kartoitus. Helsinki: Cultura Säätiö.

[ref88] Verkuyten M. J. A. M. (2009). Support for multiculturalism and minority rights: the role of national identification and outgroup threat. Soc. Justice Res. 22, 34–52. 10.1007/s11211-008-0087-7

[ref89] Vetik R. (2019). National identity as interethnic (de)mobilization: a relational approach. Ethnopolitics 18, 406–422. 10.1080/17449057.2019.1613065

[ref90] Vezzali L. Cadamuro A. Versari A. Giovannini D. Trifiletti E. (2015). Feeling like a group after a natural disaster: common ingroup identity and relations with outgroup victims among majority and minority young children. Br. J. Soc. Psychol. 54, 519–538. 10.1111/bjso.12091 25330995

[ref91] Walker I. Pettigrew T. F. (1984). Relative deprivation theory: an overview and conceptual critique. Br. J. Soc. Psychol. 23, 301–310. 10.1111/j.2044-8309.1984.tb00645.x

[ref92] Walsh S. D. Tartakovsky E. (2021). Personal value preferences, threat-benefit appraisal of immigrants and levels of social contact: looking through the lens of the stereotype content model. Front. Psychol. 12:609219. 10.3389/fpsyg.2021.609219, PMID: 33746831 PMC7970186

[ref93] Whitman D. (1998). The Optimism Gap: The I'm OK—They're Not Syndrome and the Myth of American Decline. New York: Walker.

[ref94] Wilkins C. L. Hirsch A. A. Kaiser C. R. Inkles M. P. (2017). The threat of racial progress and the self-protective nature of perceiving anti-White bias. Group Process. Intergroup Relat. 20, 801–812. 10.1177/1368430216631030

[ref95] Wright J. D. Esses V. M. (2019). It’s security, stupid! Voters’ perceptions of immigrants as a security risk predicted support for Donald Trump in the 2016 US presidential election. J. Appl. Soc. Psychol. 49, 36–49. 10.1111/jasp.12563

[ref96] Wright S. C. Taylor D. M. Moghaddam F. M. (1990). Responding to membership in a disadvantaged group: from acceptance to collective protest. J. Pers. Soc. Psychol. 58, 994–1003. 10.1037/0022-3514.58.6.994

